# Insulin Sensitivity and Pancreatic β-Cell Function in Ecuadorian Women With Turner Syndrome

**DOI:** 10.3389/fendo.2020.00482

**Published:** 2020-08-07

**Authors:** Francisco Álvarez-Nava, Daniela Bastidas, Marcia Racines-Orbe, Jéssica Guarderas

**Affiliations:** ^1^Faculty of Biological Sciences, Biological Sciences School, Central University of Ecuador, Quito, Ecuador; ^2^Institute of Biomedicine Research, Central University of Ecuador, Quito, Ecuador

**Keywords:** glucose metabolism, impaired glucose tolerance, insulin resistance indices, obesity, overweight, pancreatic β-cell function, Turner syndrome

## Abstract

**Objective:** To assess insulin sensitivity and pancreatic β-cell function in an adult population of Ecuadorian individuals with Turner syndrome (TS).

**Design and Methods:** This was a cross-sectional correlational study conducted in TS subjects (>20 years old; *n* = 38). A standard 2-h oral glucose tolerance test was performed in both women with TS and the reference group. Glucose, lipids, insulin, and C-peptide concentrations were measured. Homeostasis Model Assessment (HOMA) of Insulin Resistance, Quantitative Insulin Sensitivity Check Index, McAuley, Matsuda, and Belfiore indices were calculated to evaluate the degree of insulin resistance (IR). The pancreatic β-cell function was assessed using HOMA-β, basal C-Peptide Index (CPI), and CPII at 120′.

**Results:** A higher prevalence of impaired glucose tolerance was found in TS subjects compared with the reference group. Although significant differences were found for glucose concentrations at 60′ and 120′ (but not at 0′), only the baseline insulin concentrations differed significantly between the two groups. The values of the IR indices were statistically different between study and reference groups. A significant number of TS subjects diagnosed with IR were differently classified according to the index applied. The concentrations of C-peptide at 0′ and 120′ of TS subjects were similar to those of the control group. In contrast, the CPI and CPII values in the study group were significantly lower than those in the control group.

**Conclusion:** It is impossible to select the best surrogate method for the assessment of IR in women with TS. The CPI and CPII values could be preferable to other indices to assess the pancreatic β-cell function in TS subjects. Our findings suggest that IR and pancreatic β-cell dysfunction could be independent events in women with TS, and both conditions seem to be caused by the disease *per se*. Our results imply that early screening and intervention for TS would be therapeutic for TS women.

## Introduction

Turner syndrome (TS) is the result of a missing or structurally abnormal second sex chromosome associated with several clinical features of variable expressivity that may include dysmorphic stigmata, short stature, gonadal failure, sexual infantilism, and renal, cardiac, skeletal, and other endocrine and metabolic abnormalities. Endothelial dysfunction, impaired glucose tolerance (IGT), insulin resistance (IR), dyslipidemia, and arterial hypertension may lead to the development of type 2 diabetes mellitus (T2DM), metabolic syndrome, and cardiovascular disease (stroke and myocardial infarction) (1). The life expectancy in TS is reduced by at least 10 years due principally to a 3-fold increase in the risk of mortality from cardiovascular disease (2, 3). An altered insulin secretion appears to be a key factor of such comorbidities in TS. However, of the few studies that address insulin secretion in TS, some demonstrate hyperinsulinemia, and others suggest a decreased insulin secretion (4). Also, ~40% of adult patients with TS are affected with components of metabolic syndrome, especially dyslipidemia (5–7) and IR (8, 9), which are worsened by the coexistence of overweight/obesity (10). Nevertheless, there are conflicting results of pancreatic β-cell function and IR in women with TS (4).

The oral glucose tolerance test (OGTT) was recommended by the International Turner Syndrome Consensus Group (2017) for screening glucose homeostasis disorders in women with TS if hemoglobin A_1c_ (HbA_1c_) is elevated because they are at high risk of developing IGT (50%) due to a combination of impaired insulin secretion and IR (11). However, the best method of assessment of IR in TS subjects has not been defined. Euglycemic–hyperinsulinemic clamp technique (12) is well documented as the gold standard for assessment of IR, but it is too laborious, time-consuming, expensive, and technically complicated. The OGTT has been reported to be superior to other tests (hemoglobin A_1c_ levels, fasting or postprandial blood glucose concentrations) to detect early abnormalities of glucose metabolism in these patients (4, 13). Therefore, in this study, the objectives were to assess insulin sensitivity and pancreatic β-cell function in an adult population of Ecuadorian individuals with TS. Also, we compared the indices of IR derived from fasting concentrations values [insulin plus glucose and insulin plus triglycerides (TAGs)] such as Homeostasis Model Assessment of Insulin Resistance (HOMA-IR), Quantitative Insulin Sensitivity Check Index (QUICKI), and the McAuley index. Likewise, we wanted to contrast the aforementioned indices of IR with indices of IR derived from glucose and insulin measurements during the 75 g OGTT, such as Matsuda and Belfiore indices. Similarly, to evaluate the pancreatic β-cell function in this group of subjects with TS, we have used the HOMA-β and C-peptide indices.

## Materials and Methods

### Design Study and Study Subjects

This was a cross-sectional correlational study carried out in the School of Biology, Central University of Ecuador, Quito, Ecuador. The study protocol was reviewed and approved by the Ethics Committee for Research in Human Subjects of the Central University of Ecuador. Unrelated adult Ecuadorian TS patients (>20 years old; *n* = 38) were recruited through the Ecuadorian Foundation in Support of Turner Syndrome. They were contacted using letters, e-mails, and telephone calls and subsequently underwent a comprehensive health examination between January and November 2017. None of the subjects had received treatment with growth hormone or anabolic steroids. All except two patients had received conventional sex hormone replacement therapy (HRT). The age at which HRT was started was 17.9 ± 5 years. Of the 38 patients who constituted the study group, 21 (55%) received HRT for more than 5 years. The remaining 18 subjects were either poorly or irregularly treated (<2 years; range = 6 months to 7.3 years). Hormone replacement therapy was discontinued 4 weeks before their assessment. To ensure a comparable reference group, we prospectively recruited all TS subjects first, and then a match among reference group subjects was sought. This recruitment strategy yields 38 healthy women (20–49 years) volunteered matched by body mass index (BMI) and age. These volunteers were required to be free of significant medical illnesses and not to be taking any medication known to affect body weight or metabolic processes. All these women had regular menstrual cycles and had not been treated with or were presently receiving HRT or glucocorticoid therapy.

An appointment was given for each subject during which clinical evaluation and anthropometric measurements were performed and recorded by a single experienced physician (F. A.-N.) according to a standard protocol described elsewhere (10). Each participant was tested individually. Information regarding medical, personal, family, and dietary was obtained in the form of a questionnaire. The data collection was conducted in the morning after an overnight fast of at least 12 h. Systolic and diastolic blood pressures (SBP and DBP, respectively) were measured in each subject using an automated sphygmomanometer monitor (Omron Healthcare Inc., Bannockburn, IL, USA) on the left arm with an appropriate cuff size and in the sitting position, following 10 min of rest. Two readings at 5-min interval were obtained and averaged to determine SBP and DBP for each individual. If the two readings differed by more than 10 mm Hg, additional readings were obtained, and the last additional two readings were averaged.

### Blood Collection and Oral Glucose Tolerance Test

After a 10-h overnight fast, a total of 10 mL fasting venous blood samples for measuring serum biochemical and lipid profiles were obtained from each subject before the flavored glucose load. Serum and plasma were immediately separated. Then, a standard 2-h OGTT was performed in both TS and reference groups with 75 g glucose load at 08:00 h. Blood samples were obtained from an antecubital vein with three-point at the predetermined intervals (0, 60, and 120 min).

### Analytical Methods

The plasma glucose and serum lipids [total cholesterol (TC), high-density lipoprotein cholesterol (HDL-c), and TAGs] were immediately measured using commercially available kits with the enzymatic reference method on an autoanalyzer spectophotometer (Humalyzer 3000; HUMAN, Wiesbaden, Germany). Low-density lipoprotein cholesterol (LDL-c) was derived from the lipids measured using the Friedewald equation. The rest samples were immediately put on at room temperature, centrifuged (2,500 revolutions/min) at 4°C for 30 min to separate sera, and stored at −80°C for measurement of insulin and C-peptide. Insulin and C-peptide levels were measured by electrochemiluminescence analysis using a commercially available kit (Cobas 6000 Chemistry Analyzer; Roche Diagnostics, Indianapolis, IN, USA). Body fat assessment by bioelectrical impedance analysis was estimated using a segmental two-frequency bioimpedance analyzer (Inbody 120; Biospace Industry, Seoul, South Korea) by a trained investigator (F.A.N.) according to a standard protocol described elsewhere (10).

### Definitions

The diagnosis of TS was established by lymphocyte chromosomal analysis in combination with the presence of typical clinical features. The nutritional status classification was performed using BMI, according to the cutoffs indicated by the World Health Organization (14). Thus, all participants were classified as overweight/obese (≥25 kg/m^2^) or lean (≥18.5 < 25kg/m^2^). Essential arterial hypertension was defined by SBP ≥130 mm Hg or DBP ≥85 mm Hg or taking antihypertensive medication with no identifiable cause. Metabolic syndrome was diagnosed using the International Diabetes Federation criteria, which requires the presence of (1) central obesity (defined as waist circumference ≥80 cm) plus any two of the following four factors: (2) fasting plasma glucose 5.6 mmol/L (100 mg/dL); (3) HDL-c level <1.29 mmol/L (<50 mg/dL); (4) TAGs ≥1.7 mmol/L (>150 mg/dL) or specific treatment for this; and (5) blood pressure ≥130/85 mm Hg or treatment of previously diagnosed hypertension (15). Subjects were defined as having impaired fasting glucose (IFG) if fasting plasma glucose concentration was from 5.6 mmol/L (100 mg/dL) to 6.9 mmol/L (125 mg/dL) and/or IGT if 2-h post–glucose plasma glucose level was ≥7.8–11.0 mmol/L (140–199 mg/dL, respectively) according to the diagnostic criteria from American Diabetes Association. Subjects were divided into two groups with (a) normal glucose tolerance (NGT) (IFG and IGT negative) and (b) with IFG and/or IGT positive.

To evaluate the degree of IR, several indexes derived from either fasting or OGTT-stimulated concentrations of glucose, insulin, and TAGs were used:

1. Homeostatic Model Assessment of Insulin Resistance Index (HOMA-IR) was calculated using the equation:
  $$\begin{array}{l} \frac{\begin{array}{l} \text{fasting insulin concentration }\left(\mu \text{IU}/\text{mL}\right)\;\times \text{ fasting glucose}\\ \text{ concentration }\left(\text{mmol}/\text{L}\right) \end{array}}{22.5} \end{array}$$
2. Quantitative Insulin Sensitivity Check Index was determined according to the following formula:
  $$\begin{array}{l} \frac{1}{\begin{array}{l} \text{log fasting insulin concentration}+\text{log fasting glucose}\\ \text{ concentration} \end{array}}\; \end{array}$$
3. McAuley index was measured according to the formula:
  $$\begin{array}{l} {\text{exp}}^{\begin{array}{l} {}[2.63\;-\;0.28log\left(\text{fasting insulin concentration }\left(\mu \text{IU}/\text{mL}\right)\right)\\ -\;0.31log\left(\text{fasting triglyceride concentration }\left(\text{mmol}/\text{L}\right)\right)] \end{array}} \end{array}$$
4. Matsuda index was calculated according to the formula:
  $$\begin{array}{l} \frac{10,000}{\sqrt{\begin{array}{l} {}[\text{fasting glucose }\left(\text{mg}/\text{dL}\right)\;\times \text{fasting insulin }\left(\mu \text{U}/\text{L}\right)\\ \;\times \left[\text{MPG}\times \text{MSI during OGTT}\right] \end{array}}} \end{array}$$
  where MPG (mg/dL) is mean plasma glucose OGTT, and MSI (μIU/mL) is mean serum insulin during OGTT.
5. Belfiore index was calculated from changes in plasma glucose and serum insulin using the equation:
  $$\begin{array}{l} \frac{2}{\left(1/\left(\text{AUC insulin }\times \text{ AUC glucose}\right)\right)\;+\;1}\; \end{array}$$

where AUC is the values of the area under curve insulin (μIU/mL) and glucose (mmol/L) concentrations in the course of OGTT, respectively.

The pancreatic β-cell function was evaluated using the formulas of Hovorka et al. with some modifications.

1. The HOMA-β index, which was calculated using the following equation:
  $$\begin{array}{l} \frac{20\;\times \text{ fasting insulin }\left(\text{mU}/\text{mL}\right)}{\left(\text{fasting plasma glucose }\left(\text{mmol}/\text{L}\right)-3.5\right)} \end{array}$$
2. The C-Peptide Index (CPI), which represents the ability of fasting glucose to stimulate pancreatic β-cell secretion (fasting pancreatic β-cell responsiveness), was determined according to the following formula:
  $$\begin{array}{l} \frac{100\;\times \text{ fasting C}-\text{peptide }\left(\text{ug}/\text{L}\right)}{\text{fasting glucose concentration }\left(\text{mg}/\text{dL}\right)} \end{array}$$
3. The CPII index, which represents the ability of postprandial glucose to step up pancreatic β-cell secretion, was calculated by
  $$\begin{array}{l} \frac{\begin{array}{l} 100\;\times \;[120^{\prime }\text{ C}-\text{peptide}\left(\text{ug}/\text{L}\right)\text{ during OGTT }-\text{fasting C}\\ \;-peptide\;\left(\text{ug}/\text{L}\right)] \end{array}}{\begin{array}{l} 120^{\prime }\text{ glucose concentration }\left(\text{mg}/\text{dL}\right)\text{during OGTT }-\text{fasting}\\ \text{ glucose concentration }\left(\text{mg}/\text{dL}\right) \end{array}} \end{array}$$

According to different indices, the following thresholds defined IR state among study and reference group subjects: HOMA-IR ≥ 2.5, QUICKI ≤0.339, McAuley index ≤ 5.8, Matsuda index ≤ 4.6, and Belfiore index ≥1.23 (16–18).

### Statistical Analysis

Data were analyzed using IBM SPSS Statistics 19.0 (SPSS Inc., Chicago, IL, USA) and GraphPad InStat 7.00 (GraphPad Software, Inc., La Jolla, CA, USA). The general features of the participants are described as number of cases and mean and standard deviations unless otherwise mentioned. Results as determined by 95% confidence intervals (95% CIs) and *P* < 0.05 were considered to be statistically significant. The prevalence of metabolic syndrome, overweight/obesity, IGT, and IR were indicated as percentages. To compare the proportions of categorical variables, the two-proportion *Z* test was used. After checking the normality of the quantitative variables with the Kolmogorov–Smirnoff or the Shapiro–Wilk tests (*P* < 0.05), a Student *t*-test was performed to compare means between groups for continuous variables. Conversely, when the variables did not fit into the criteria of normality, they were expressed as median (range), and a Mann–Whitney *U* test was carried out. Likewise, the association between quantitative variables was examined using Pearson or Spearman correlation analysis depending on the distribution assumed by the data. The degree of agreement or concordance between the IR indices derived from fasting concentration values and glucose and insulin levels during OGTT was reported by using the Cohen κ coefficient with 95% CIs, with levels of agreement interpreted according to Landis and Koch (19), κ < 0.40, 0.41–0.60, 0.61–0.80, and 0.81–1.00 to considered fair, moderate, substantial, and almost perfect agreement, respectively. Bivariable linear regression analysis was performed to identify independent influencing factors for pancreatic β-cell function in TS subjects by a marginal statistical significance *P* < 0.2. These independent variables (age; waist circumference; fat mass; glucose and insulin concentrations at 0′, 60′, and 120′; and HOMA-IR) were considered candidate variables and exported to a multiple linear regression model with HOMA-β as the dependent variable to assess the magnitude of their individual effect on the pancreatic β-cell function and for controlling the possible effect of confounders. In the final multivariable model, *P* < 0.05 for two-sided tests was set as the level of significance.

## Results

### Clinical, Anthropometric, and Biochemical Features of the Study Group and Reference Groups

The anthropometric and metabolic variables of TS and reference groups are described in Table 1. Although no significant difference was found for BMI between both groups, weight and waist circumference, TAGs, TC, LDL-c, fat mass, percentage of fat mass, and the free fat mass–to–fat mass ratio in the study group were significantly greater than those in the reference group.

**Table 1:** Clinical and metabolic features of TS subjects and reference group.

	**TS Subjects**	**Reference Group**	***P*-value**
*n*	38	38	
Age (years)	29.23 ± 8.09	29.81 ± 4.7	0.936
Height (cm)	139 ± 0.04	148 ± 0.01	<0.0001
Weight (kg)	51.01 ± 14.14	54.08 ± 9.08	0.0406
BMI (kg/m^2^)	26.17 ± 7.41	24.72 ± 3.89	0.813
Triglycerides (mg/dL)	158.9 ± 67.75	117.87 ± 58.06	0.0071
Total cholesterol (mg/dL)	245.90 ± 38.13	180.99 ± 23.39	<0.0001
LDL-c (mg/dL)	203.69 ± 60.43	101.71 ± 19.64	<0.0001^*^
HDL-c (mg/dL)	60.68 ± 17.73	55.70 ± 13.19	0.4508
Glucose 0′ OGTT (mg/dL)	81.68 ± 10.28	79.36 ± 6.93	0.3088^*^
Glucose 60′ OGTT (mg/dL)	132.65 ± 39.91	90.71 ± 1.06	<0.0001^*^
Glucose 120′ OGTT (mg/dL)	118.50 ± 33.46	85.26 ± 15.37	<0.0001^*^
Insulin 0′ OGTT (μU/mL)	12.09 ± 8.62	9.18 ± 6.42	0.008
Insulin 60′ OGTT (μU/mL)	90.06 59.85	74.29 ± 54.09	0.1393
Insulin 120′ OGTT (μU/mL)	85.57 ± 56.70	78.24 ± 75.03	0.3994
C-peptide 0′ (ng/mL)	2.58 ± 1.34	1.99 ± 0.97	0.0528
C-peptide 60′ (ng/mL)	8.66 ± 3.12	7.17 ± 2.31	0.0369
C-peptide 120′ (ng/mL)	9.20 ± 3.39	7.16 ± 2.9	0.0963
HOMA-IR	2.57 ± 2.09	2.18 ± 1.22	0.8476
HOMA-β	265.18 ± 83.74	332.38 ± 42.40	0.7227
Matsuda index	5.57 ± 3.72	6.14 ± 3.39	0.3671
QUICKI index, % (*n*)	0.35 ± 0.04	0.34 ± 0.03	0.7695^*^
Metabolic syndrome	42.1 (16) (0.2–0.5^§^)	13.33 (4) (0.05–0.3^§^)	<0.05^***^
Overweight/obesity	42.11 (16) (0.28–0.61^§^)	50 (15) (0.33–0.67^§^)	>0.05^**^
Insulin resistance	33.33 (10) (0.2–0.5^§^)	33.33 (10) (0.2–0.5^§^)	>0.05^**^
Impaired glucose regulation	26.6 (8) (0.1–0.44^§^)	0 (0.0–0.113^§^)	<0.05^**^

* *Student t-test*.

** *Two-proportion Z test*.

§ *95% confidence intervals. Insulin Resistance is defined by HOMA-IR = ≥ 2.5. BMI, body mass index; HDL-c, high-density lipoprotein cholesterol; LDL-c, low-density lipoprotein cholesterol; OGTT: oral glucose tolerance test, TC, total cholesterol*.

The prevalence of overweight/obesity was similar between both groups (two-proportion *Z* test *P* > 0.05, 95% CI = −0.18 to 0.3). Although the prevalence of IR (according to HOMA-IR) was similar between both groups (two-proportion *Z* test *P* > 0.05, 95% CI = 0.34–2.92), a higher prevalence of IGT was found in TS subjects compared with the reference group (two-proportion *Z* test *P* < 0.05, 95% CI = 0.428–0.105). Significant differences for fasting glucose (*P* < 0.022) and insulin (*P* < 0.004), and HOMA-IR (*P* < 0.005), Matsuda (*P* < 0.004), and QUICKI (*P* < 0.001) indexes were found between TS women when they were categorized according to their nutritional status (<25 or ≥25 kg/m^2^). The concentrations of C-peptide at 0′ and 120′ of TS subjects were similar to those of the control group (Table 1). In contrast, the CPI values in the study group were significantly lower than those in the control group [median = 0.89 (range = 0.67–1.56) vs. 1.53 (0.89–1.98), *P* = 0.01]. Similarly, the values of CPII in the study group were significantly lesser than those in the reference group [median = 1.57 (range = 0.67–1.56) vs. 5.23 (0.89–1.98), *P* = 0.0001, 95% CI = 1.093–20.7].

During OGTT, the maximum glucose and insulin concentration peak was found at 60′ for both the study group and the reference group. Although the study group maintained higher glucose and insulin values than the reference group over time (0′, 60′, and 120′), there were only significant differences for glucose concentrations at 60′ and 120′ (*P* < 0.001). Also, TS subjects with overweight/obesity (≥25 kg/m^2^) showed higher glucose values at 60′ and 120′ than those found for lean TS individuals (*P* < 0.0001) or the two reference subgroups (*P* < 0.0001). When the four subgroups (subcategorized according to BMI) were compared, baseline glucose concentrations did not differ significantly between the four subgroups. However, significant differences were found in the plasma glucose concentration at 60′ and 120′ between the different subgroups analyzed (*P* < 0.0001). In contrast, although baseline serum insulin concentrations between the two subgroups of individuals with TS differed significantly, insulin levels at 60′ and 120′ between the four subgroups were similar. As expected, both in the study and reference groups, there was a significant correlation between the general adiposity variables (weight and BMI) and IR indices. However, HOMA-β weakly correlated (weight) or not correlated (BMI) with these anthropometric adiposity variables (*r* = 0.38; *P* < 0.037; *r* = 0.34; *P* < 0.063, respectively) in TS subjects.

### Correlation Between Different Insulin Resistance Indices in the TS Group

The values of 60′ or 120′ glucose concentrations during the OGTT were correlated positively with HOMA-IR (*r* = 0.35, *P* < 0.032, and *r* = 0.397, *P* < 0.021, respectively) and negatively with the Matsuda index (*r* = −0.512, *P* = 0.004, and *r* = 0.478, *P* < 0.008, respectively) in TS subjects. Spearman rank correlations between IR indices derived from fasting values of glucose and insulin and/or TAGs and OGTT in TS subjects are presented in Table 2. Among the former, a high degree of correlation between HOMA vs. QUICKI, (*r* = −0.994, *P* < 0.0001) and HOMA-IR vs. McAuley (*r* = −0.835) was detected. In addition, a moderate correlation between OGTT-derived IR indices (Belfiore vs. Matsuda index, *r* = −0.748) was also observed. By contrast, the correlation between IR indices based on fasting values and OGTT was highly variable, ranging from high correlation between HOMA-IR and Matsuda index (*r* = −0.953, *P* < 0.0001) and QUICKI and Matsuda index (*r* = −0.959, *P* < 0.0001), through moderate correlation between McAuley and QUICKI (*r* = 0.784), HOMA-IR, and Belfiore (0.745) or Belfiori vs. QUICKI (*r* = −0.692).

**Table 2:** Correlation between insulin resistance indices in subjects with Turner syndrome.

	**HOMA-IR**	**QUICKI**	**McAuley index**	**Matsuda index**	**Belfiore index**	**HOMA-β**
HOMA-IR	—	−0.9937	−0.803	−0.9531	−0.811	0.4342
QUICKI	<0.0001	—	0.792	0.9599	0.692	−0.4165
McAuley index	<0.0001	<0.0001	—	0.750	0.626	−0.148
Matsuda index	<0.0001	<0.0001	<0.0001	—	0.917	−0.4136
Belfiore index	<0.0001	<0.0001	<0.0001	<0.0001	—	−0.403
HOMA-β	0.0165	0.022	0.434	0.023	0.027	—

*Spearman correlation coefficients are above the diagonal; P-values for each pairwise correlation are below the diagonal*.

The prevalence of IR was highly variable in the study group according to HOMA-IR (42.1%), QUICKI (44.7%), Belfiore (51.2%), or Matsuda (55.3%) indices. A broad range of concordance was found between fasting or OGTT-stimulated indices. Thus, if IR was defined according to HOMA-IR (cutoff = ≥2.5), 97.4% (16/17, κ = 0.946) of the subjects in the study group had also IR as reported by QUICKI (cutoff = ≥0.34), considered as an almost perfect agreement. Besides, if IR was considered according to the Matsuda index (cutoff = ≥4.6), 88.9% (16/18, κ = 0.918) of TS individuals was also IR as stated in the Belfiore index (cutoff = 1.23). Conversely, a lower but substantial concordance was found when the IR indices based on fasting glucose and insulin concentrations were contrasted with those based on the OGTT concentrations. For example, 76.19% (16/21) of TS subjects with IR according to the HOMA-IR were not considered IR if the Matsuda index was applied (κ = 0.741).

### Insulin Sensitivity vs. Pancreatic β-Cell Function in TS Subjects

In TS subjects, HOMA-IR values showed a wide range of IR from 0.3 to 7.2. Similarly, HOMA-β values ranged from 33.7 to 525.6% in TS subjects, indicating a broad range of insulin secretion. A significant (*P* < 0.018) but only moderate correlation (*r* = 0.43) between HOMA-IR and HOMA-β was found in the study group. Thus, elevated IR with an increasing (compensatory) insulin secretion was demonstrated. However, in the TS subject group, this correlation was lower (*r* = 0.429) than that observed in the reference group (*r* = 0.487, *P* < 0.0006).

Because the pancreatic β-cell function may be influenced by a variety of factors, a multiple linear regression analysis was performed with HOMA-β as the dependent variable and age, height, weight, BMI, fat mass, the HOMA-IR index, and glucose and insulin concentrations at 0′, 60′, and 120′ as independent variables. Because of a strong correlation between HOMA-IR and QUICKI indices (*r* = −0.994) in TS subjects, only HOMA-IR was added to this model as an IR index. The model explained a part of the variance of the HOMA-β with adjusted *R*^2^ values of 0.687. Approximately only 20% of the total variation in HOMA-β can be explained by the HOMA-IR index (*R*^2^ = 0.192, *P* < 0.02). Also, on stepwise multivariable regression, HOMA-IR and glucose concentration at 0′, 60′, and 120′ were independent factors for HOMA-β in TS subjects, but after adjusting the model for fasting glucose concentration, the influence of HOMA-IR on HOMA-β decreased [standardized regression coefficient (β) = 0.237, *P* < 0.0487].

## Discussion

Adult TS subjects are susceptible to IGT and T2DM (8, 20–22). The prevalence of IGT in TS patients is ~10 to 34% (4), which is higher than that in the healthy population. In a study of 103 TS subjects, the prevalence of IGT and T2DM was reported in 7.48 and 1.9%, respectively (5). In our study, IGT was found in 18 and 38% of lean and overweight/obese TS individuals, respectively, with a global prevalence of 27%. No individual in the reference group had glucose metabolism abnormalities. Subjects with T2DM were excluded from our study. Thus, a higher prevalence of IGT was observed in the present study compared to those previously reported in the literature. This may be due to the different age ranges, ethnic origin, and a higher BMI reported in our study. We included only TS subjects ≥20 years of age in our study. In studies in TS subjects with a wide age range, the prevalence of IGT is lower. Our reference group was age- and BMI-matched with the study group. Although overweight/obesity is a well-known risk factor for IRG, BMI did not explain the higher glucose levels during OGTT found in our study group compared with the reference group.

Our results suggest that the HOMA-IR and Matsuda indices may represent a good alternative combination to the euglycemic–hyperinsulinemic clamp in the assessment of IR in TS. Because of a good correlation between these two indices (*r* = −0.748), it is reasonable to think that this combination is the best instrument to evaluate IR in TS. However, some caution should be considered. The McAuley index had a strong linear correlation with both HOMA-IR and QUICKI, suggesting that it can be used together with other indices to assess IR in TS subjects in a clinical context. Also, the Belfiore index and HOMA-IR had similar correlations with the Matsuda index (r= −0.744 and 0.748, respectively). In addition, the presence of a good correlation does not necessarily mean that HOMA-IR and Matsuda indices have the best performance predictive in diagnosing IR in our TS patients because there were some borderline values in a relatively small group of TS individuals in our study. Additionally, the correlation coefficients of the Matsuda index with QUICKI (*r* = 0.9599 vs. −0.954) and the McAuley index (*r* = 0.795 vs. 0.758) are stronger than those observed with HOMA-IR, which were also calculated from fasting concentrations. Finally, varying correlation and agreement were found between IR indices based on fasting concentrations and those calculated on OGTT values. Consequently, the assessment of IR in TS subjects produced significantly different results according to the method applied (fasting vs. OGTT concentrations). Furthermore, our findings revealed that a significant number of our TS patients were differently classified according to the indices applied. Thus, it is impossible to select the best surrogate method for the assessment of IR in women with TS. Therefore, the utilization of any surrogate IR indices in the clinical scenario of TS must be viewed with extreme caution.

Several studies have examined the sensitivity or secretion of insulin in TS. Insulin resistance is clearly found in many subjects with TS (8, 9, 23). However, different results have been reported, depending on whether insulin sensitivity was assessed from the fasting concentration or in the postabsorptive state. On the other hand, whereas some studies have demonstrated a decreased insulin secretion (9, 24), other studies have reported an increased insulin secretion in TS compared with normal subjects, probably as compensation for IR (25, 26). Thus, although several studies have examined insulin sensitivity or pancreatic β-cell function in TS, thus far, the results are equivocal. A possible confounding factor is that these studies fail to adjust insulin secretion to the prevailing degree of IR. Although compensatory hyperinsulinemia may be detected, it may not be appropriately elevated to match the degree of IR. Also, insulin secretion and insulin sensitivity have been shown to have a hyperbolic association (27). Therefore, it is necessary to adjust the insulin secretion for IR to accurate interpretation of the pancreatic β-cell function. In contrast to several studies examining the sensitivity or secretion of insulin in TS, to the best of our knowledge, this is the first study that explores the relationship between insulin sensitivity (HOMA-IR) and pancreatic β-cell function (HOMA-β) in TS subjects. We tested the association between HOMA-IR and HOMA-β rather than comparing mean values. Because the pancreatic β-cell function, measured by HOMA-β, may be influenced by a variety of factors, we performed a multiple linear regression analysis with the variables showing correlation (age, waist circumference, fat mass, fasting glucose, and insulin concentrations at 0′, 60′, and 120′, HOMA-IR). Overall, the model explained a large part of the variance of the pancreatic β-cell function with adjusted *R*^2^ values of approximately 0.7. However, the influence of BMI was not significant in the model. Also, the model remained explaining much of the variance of the pancreatic β-cell function when it was adjusted by HOMA-IR.

As the BMI does not seem to be an independent factor of the pancreatic β-cell function in our study, we did find an increased but insufficient insulin secretion both in lean and overweight/obese TS subjects in our study. These findings reinforce the idea that despite having a compensatory response to glucose overload during OGTT, insulin secretion is not enough to maintain adequate glucose levels at 60 and 120 min. Thus, a higher IR with a lower insulin secretion seems to indicate a declining pancreatic β-cell function in our TS subjects. We cannot prove whether these findings are a reflection of an evaluation carried out at an early stage of the disorder, because the study group was represented by women with 29.23 ± 8.09 years. However, our findings suggest that a decrease in insulin secretory response observed in our TS subjects is an underlying mechanism that can lead to T2DM in a short time.

In the clinical setting, HOMA-β and insulinogenic indices are usually used to measure the pancreatic β-cell function (28, 29). However, the insulin reserve cannot be accurately estimated on the insulin levels because of its pulsatile release pattern and short half-life. The pancreatic β-cell area has been reported to correlate with the C-peptide–to–glucose ratio calculated from the concentrations of 75 g OGTT but not with HOMA-β (30). C-peptide is produced in equimolar amounts to endogenous insulin, but unlike insulin, this is subject to neither hepatic nor significant peripheral degradation as is mainly removed by the kidneys. Thus, C-peptide can be used to assess the pancreatic β-cell function. The increment of C-peptide immunoreactivity level in the glucagon test is probably the most accurate test to evaluate insulin reserves as has the advantage of being a more reproducible stimulus and having much faster action on pancreatic β cells (31). However, the OGTT has the advantage of being a better physiological stimulus, avoiding the side effects of glucagon. Besides, compared with the fasting C-peptide level, the C-peptide level at 120′ during OGTT is more capable of representing the maximal pancreatic β-cell secretory capacity (32). In our study, the metabolic variables representing the ability of fasting glucose to stimulate β-cell secretion, such as fasting C-peptide, postprandial insulin, and C-peptide, were similar between the study and reference groups. However, the CPI and CPII indices indicating the ability of postprandial glucose to step up pancreatic β-cell secretion differed significantly between the two groups. This seems to indicate that the higher levels of basal insulin in our TS subjects represent a diminution of peripheral insulin sensitivity rather than a conserved function of the pancreatic β-cells.

The observed variability in correlation coefficients between insulin sensitivity indices calculated from the fasting concentration and those based on OGTT in women with TS led us to ask whether the relationship between HOMA-IR and HOMA-β (a good measure of pancreatic β-cell function) was different between TS and reference subjects. A Spearman correlation showed a significant but moderate association between HOMA-IR and HOMA-β both TS and reference subjects (0.429 vs. 0.487, respectively). The lower effect of HOMA-IR on HOMA-β in our TS subjects indicates that a given increase in HOMA-IR leads to a lesser raising in HOMA-β in TS subjects than in the reference group. Although this difference is minimal, it should also be considered that a significant difference between plasma concentrations of glucose at 60′ and 120′ was found between both groups and that the serum concentrations of insulin during OGTT were similar between the study and reference groups. Thus, all these findings suggest that the pancreatic β-cell function is inadequate. Therefore, our TS individuals seem to have an increased risk of the pancreatic β-cell exhaustion and the development of T2DM. Type 2 diabetes mellitus develops when the pancreatic β cells become “exhausted” and cannot secrete adequate amounts of insulin to preserve normoglycemia in an increasing IR environment. All these altered mechanisms seem to be present in our TS subjects.

Circulating insulin levels at 60′ and 120′ were similar when our TS subjects were compared according to their glucose metabolism status. However, significant differences were found in the plasma glucose concentration at 60′ and 120′ between our TS subjects with or without IGT. This contrasts with the findings in glucose concentration during OGTT in our TS subjects when they were discriminated by BMI. In this scenario, only a significant difference in basal glucose concentration was found between both groups. In addition, TS subjects with IGT showed basal circulating insulin levels similar to those found in NGT-TS subjects. Furthermore, no significant difference was found in serum levels of insulin at 60′, and 120′ during OGTT between lean TS women and overweight/obese TS subjects. Thus, in our TS subjects, an NGT or lean status did not mean that insulin secretion was normal. This probably reflects early IR, and plasma glucose concentration is maintained to a certain degree. Not only do all these findings reflect a lower insulin sensitivity in our TS individuals, but also increased insulin levels are not enough to maintain normal glucose homeostasis. Thus, IR and β-cell dysfunction could be independent events in women with TS, and both conditions seem to be caused by the disease itself. This may be the result of deletions of some genes related to insulin signal transduction and pancreatic β-cell function located on the X chromosome. Bakalov et al. (21) observed that the long arm of the X chromosome (iXq) is associated with a higher incidence of T2DM as compared to the 45,X group. A proposed explanation for this increased risk could be that haploinsufficiency for unknown Xp gene(s) constitutes a “first hit” that causes the basic deficit in pancreatic β-cell function seen in 45,X patients. Excess dosage of Xq genes in isochromosome Xq may provide a “second hit” that exacerbates the deficit, perhaps by altering other genes involved in pancreatic β-cell development and function or survival, and/or by stimulating low-grade chronic autoimmunity that injures but does not obliterate the pancreatic β-cells. However, so far, such mechanisms have not been demonstrated, and potentially involved genes have not been identified. An alternative hypothesis is that both conditions (IR plus pancreatic β-cell dysfunction and TS) are comorbidities caused by a common factor. The cell cycle delay hypothesis states that a retarded cell cycle due to aneuploidy will reduce the number of cell cleavages per time units (33). Therefore, there will not be enough time for pancreatic β cells to develop fully and to grow to their optimal function. Also, a retarded cell cycle might origin a disturbed intracellular metabolism in general as a result of unbalanced gene content. No association was found between IR or pancreatic β-cell dysfunction and karyotype in our study.

Several limitations of our study should be considered. Sufficiently large sample size will be needed to reach reliable conclusions. Also, the cross-sectional nature of our study prevents us from delineating pathogenic interpretations of our findings. A longitudinal evaluation of the capacity of IR indexes to predict β-cell decompensation in adult TS individuals is warranted. Although statistically significant differences were determined between the TS subjects and the reference group and between NGT-TS and IGT-TS, caution should be considered. We applied the Kruskal–Wallis test with *post hoc* correction to multiple comparisons to reduce the chances of obtaining a type I error. Also, the TS group with IGT was small, which prevented us from detecting relevant features of this group. Besides, we were unable to establish cutoff points by percentiles for defining IR to each index. We cannot apply percentile distribution for IR indices derived from other populations (e.g., Caucasian) as this may be very different because our data have been obtained from an entirely Latin American population, and a broad variability in the concordance analysis of selected IR indices was found in our study. As well, we cannot generalize the conclusions to other ethnic populations. Lastly, although euglycemic–hyperinsulinemic clamp and the intravenous glucose tolerance test with minimal modeling techniques are more accurate than OGTT, this latter is much more physiological than the intravenous tests, mainly because glucose sensors disseminated by the gastrointestinal tract take part in insulin secretion (34). In addition, studies have shown that OGTT is superior to other tests for the diagnosis of early abnormalities of glucose metabolism in these patients (4, 11, 13).

In summary, a broad variety of correlation coefficients between methods based on fasting data of glucose and insulin vs. methods of OGTT-derived values was found in our study. Consequently, adult TS subjects diagnosed with IR by one method might not have equally IR if diagnosed by another method. Therefore, it is impossible to select the best surrogate method for the assessment of IR in women with TS. Based on our findings of OGTT, CPI and CPII values could be preferable to other indices to assess the pancreatic β-cell function in TS patients in a clinical setting. Lastly, an impaired β-cell function was observed in our cohort of TS subjects both in lean and overweight women with TS, as estimated by adjusting for IR. Our findings suggest that the compromised pancreatic β-cell function in TS subjects might differ from that seen in obese women without TS. Thus, an important question raises whether TS *per se* could account for the development of IGT or T2DM. Because this defect was not associated with BMI in our patients, it may be caused by intrinsic factors of TS. Our findings imply that early screening and intervention for TS would be therapeutic for TS women.

## Data Availability Statement

The datasets generated for this study are available on request to the corresponding author.

## Ethics Statement

The studies involving human participants were reviewed and approved by Subcomité de Ética de Investigación en Seres Humanos de la Universidad Central del Ecuador. The patients/participants provided their written informed consent to participate in this study.

## Author Contributions

FÁ-N: study concepts and design, data analysis and interpretation, statistical analysis, obtaining funding, critical revision of the manuscript for important intellectual content, and manuscript preparation. DB: data analysis and interpretation, statistical analysis, and critical revision of the manuscript for important intellectual content. MR-O: biochemical studies and data analysis. JG: statistical analysis. All authors contributed to the article and approved the submitted version.

## Conflict of Interest

The authors declare that the research was conducted in the absence of any commercial or financial relationships that could be construed as a potential conflict of interest.
